# Bistability of AlGaAs/GaAs Resonant-Tunneling Diodes Heterostructural Channel

**DOI:** 10.3390/s23187977

**Published:** 2023-09-19

**Authors:** Natalia Vetrova, Evgeny Kuimov, Vladimir Sinyakin, Sergey Meshkov, Mstislav Makeev, Vasiliy Shashurin

**Affiliations:** Research Institute of Radio Electronics and Laser Technology, Bauman Moscow State Technical University, 105005 Moscow, Russia; ekjmo@mail.ru (E.K.); v.sinyakin@gmail.com (V.S.); meschkow@bmstu.ru (S.M.); m.makeev@bmstu.ru (M.M.); schashurin@bmstu.ru (V.S.)

**Keywords:** mathematical modeling, resonant-tunneling structures, hysteresis, self-consistent potential, resonant levels, semiconductor epitaxial layers, radio frequency converting devices, energy efficiency, smart city, industry 4.0, information and communications technology for development

## Abstract

This paper presents an effective compact model of current transfer for the estimation of hysteresis parameters on the volt-ampere characteristics of resonant-tunneling diodes. In the framework of the compact model, the appearance of hysteresis is explained as a manifestation of internal bistability due to interelectronic interaction in the channel of the resonant-tunneling structure. Unlike the models based on the method of equivalent circuits, the interelectronic interaction in the compact model is taken into account using the concentration parameter. Model validation allowed us to confirm the high accuracy of the model not only at the initial section of the volt-ampere characteristics, but also at the hysteresis parameters traditionally predicted with low accuracy, namely the loop width (∆ < 0.5%) and contrast (∆ < 7%). Thus, it is concluded that the models are promising for integration into systems for synthesizing the electrical characteristics of resonant-tunneling diodes.

## 1. Introduction

Sensor networks based on radio frequency identification (RFID) technology with passive tags do not require a built-in power source. This makes these sensors promising for use in hard-to-reach areas, such as the Arctic and Antarctica, or in space, as well as for implantable biosensors used in biotelemetry and telemonitoring.

The authors have shown that the use of resonant-tunneling diodes (RTDs) in the rectifier of a passive RFID tag system allows for increased sensitivity and, as a result, a 2–3-fold increase in the range of wireless sensor networks based on RFID technology with passive tags, or a reduction in the harmful effects of electromagnetic radiation from the reader device [1,2,3]. This is relevant for biomedical applications (including the concept of “smart city”), as well as for improving the electromagnetic compatibility of devices within the network’s range by reducing the reader’s radiation power. In addition, the attractiveness of RTDs as nonlinear elements of radio-electronic sensors is due to the possibility of increasing the operating frequencies of these devices up to the terahertz range [4,5,6,7].

The attractiveness of resonant-tunneling diodes as a nonlinear element is primarily due to the possibility of targeted synthesis of their voltage–current characteristics (I-V curves) through optimal design implementation of the resonant-tunneling structure (RTS) to achieve the required level of converter signal parameters during the design stage [8,9,10,11,12,13,14]. Thanks to the variation in I-V curves, research on the development of a “universal” resonant-tunneling diode capable of functioning as both a generator and a detector depending on the bias voltage is considered promising. To solve this problem, as well as for I-V curve synthesis for specialized applications, there is a need to optimize the design of resonant-tunneling diodes.

However, nowadays, the task of designing a converter with the required parameters is not completely solved. Existing I-V curve models are not effective in synthesizing the RTS based on the criteria of the desired level of signal converter performance for the following reasons:

1. Reasonable accuracy of I-V curve prediction is achieved within a narrow voltage range (it should be noted that this severely limits the practical application spectrum of RTDs, especially for sources and detectors of terahertz radiation).
2. Inadequate modeling of non-stationary processes in the RTS and, as a result, the inability to predict hysteresis phenomena in RTD I-V curves.
3. The high temporal and spatial complexity of existing computational algorithms for I-V curves, considering dissipative processes in the RTS, leads to the fundamental unsolvability of the optimization problem for the design synthesis of RTDs (the problem belongs to the class of NP-complete problems) for signal converters with the required level of performance parameters.

Thus, the implementation of the advantages of RTDs as nonlinear elements in modern sensors is impossible without the development of a practice-oriented model (efficient and compact in terms of temporal and spatial complexity criteria) that allows not only for qualitative analysis of current transport processes but also for obtaining quantitative assessments of operational parameters of RTDs and devices based on them, including considering technological factors.

## 2. Modeling Methodology

### 2.1. Main Relationships of the Effective Stationary Compact Model

Let us consider the main equations of the combined self-consistent model of current transport in heterostructures with transverse transport [15,16,17,18], which is based on the formalism of envelope wave functions, which are solutions of the Schrödinger equation for an open system. The advantages of this model are its high level of validation at the initial part of the RTD I-V curve and its high degree of elaboration, which make it a suitable “verification basis” for building a compact model.

The main goal of modeling charge transport phenomena is to calculate the fluxes and concentrations of charge carriers (in the considered problem, electrons). The fluxes and concentrations of electrons in the combined model are considered as the sum of fluxes and concentrations at all available energy levels, for which the well-known density of states apparatus is used. Therefore, the current and electron concentration in the considered model are determined as follows:

(1) $$\begin{array}{c} J\left(V\right)=qS_{m}\int_{U_{s}}^{\infty }\rho_{s}\left(z_{d}\right)v_{s}f_{2D}\left(E-U_{s}\right)dE-qS_{m}\int_{U_{d}}^{\infty }\rho_{s}\left(z_{d}\right)v_{d}f_{2D}\left(E-U_{d}\right)dE\\ n\left(z\right)=\int_{U_{s}}^{\infty }\rho_{s}\left(z\right)f_{2D}\left(E-U_{s}\right)dE+\int_{U_{d}}^{\infty }\rho_{d}\left(z\right)f_{2D}\left(E-U_{d}\right)dE \end{array},$$

where $U_{s\left(d\right)}$ is the potential energy of electrons in the source (drain), E is the energy of electrons, q is the elementary charge, ℏ is the Dirac constant, m is the effective mass of electrons in the reservoirs, $\rho_{s\left(d\right)}$ is the local density of states (LDOS) of electrons in the source (drain), $v_{s\left(d\right)}$ is the absolute value of the velocity of electrons in the reservoirs in the source (drain), $f_{2D}\left(E\right)$ is the two-dimensional distribution function of electrons, $S_{m}$ is the area of the mesa, $z_{s\left(d\right)}$ is the coordinate of the source–channel (channel–source) boundary, V is the external voltage.

It should be noted that LDOS and the electron flux density $j_{s\left(d\right)}=\rho_{s\left(d\right)}v_{s\left(d\right)}$ are related by the continuity equation and are determined by the following formulas:

(2) $$\begin{array}{c} \rho_{s\left(d\right)}=\frac{m}{\pi \hbar^{2}}\frac{{\left|\psi_{s\left(d\right)}\right|}^{2}}{\left|k_{s\left(d\right)}\right|}\\ v_{s\left(d\right)}=\frac{\hbar \left|k_{s\left(d\right)}\right|}{m} \end{array},$$

where $\psi_{s\left(d\right)}$ represents the wave functions of the source (drain) electrons, $k_{s\left(d\right)}$ represents the wave numbers of the source (drain) electrons in the respective reservoirs.

The wave numbers of the electrons and their two-dimensional distribution functions are defined as follows:

(3) $$\begin{array}{c} \left|k_{s\left(d\right)}\right|=\frac{\sqrt{2m\left(E-U_{s\left(d\right)}\right)}}{\hbar }\\ f_{2D}\left(E\right)=\frac{mkT}{2\pi \hbar^{2}}\ln\left(1+e^{\frac{E_{F}-E}{kT}}\right) \end{array}$$

where $E_{F}$ is the Fermi level in reservoirs.

The main idea of developing a compact model is that the system of integral and differential equations of the verification basis, according to the basic principles of linearization and decomposition, can be transformed into algebraic and transcendental equations. These methods are based on splitting the system of coupled equations into several simpler independent equations. Decomposition significantly simplifies the qualitative analysis and interpretation of important physical properties described by the coupled equations, effectively allowing the study of their wave and dissipative properties. Moreover, in some cases, decomposition enables finding exact analytical solutions to the corresponding boundary value and initial boundary value problems and greatly facilitates the application of numerical methods.

To build a compact model, let us perform the decomposition of Equation (1). It should be noted that we will only consider the concentration of electrons in the center of the quantum well of the RTS since it will be associated with the energy of resonance levels. In the stationary case, the continuity equation implies that the electron flux density is constant, allowing us to write

(4) $$\rho_{s\left(d\right)}\left(z_{d\left(s\right)}\right)v_{d\left(s\right)}=j_{s\left(d\right)}\left(z_{d\left(s\right)}\right)=j_{s\left(d\right)}\left(z_{w}\right)=\rho_{s\left(d\right)}\left(z_{w}\right)v_{w},$$

where $z_{w}$ is the coordinate of the center boundary of the well, $j_{s\left(d\right)}\left(z_{d\left(s\right)}\right)$ is the electron flux density, $v_{w}$ is the electron velocity in the quantum well.

**Figure 1:**
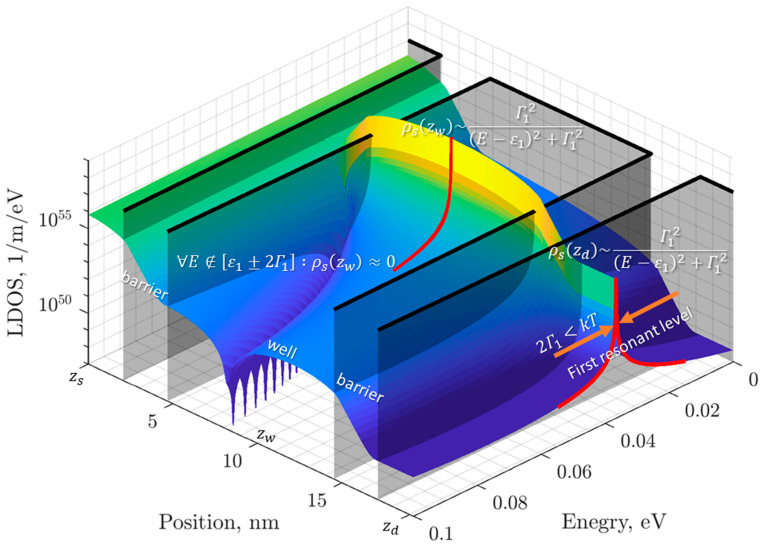
The illustration of model assumptions of the first resonant level as an example, black surface is for profile of a conduction band bottom.

A common way to simplify models of current transport is to use the approximation of the tunneling transparency coefficient in the vicinity of the resonance level when $E\in \varepsilon_{n}\pm \Gamma_{n}$ with a Lorentz function [19] (Figure 1, red curve):

(5) $$T\left(E\right)={\left|\psi_{s\left(d\right)}\left(z_{d\left(s\right)}\right)\right|}^{2}\frac{\left|k_{d\left(s\right)}\right|}{\left|k_{s\left(d\right)}\right|}\approx \overset{N}{\underset{n=1}{\sum }}\frac{\Gamma_{n}^{2}}{{\left(E-\varepsilon_{n}\right)}^{2}+\Gamma_{n}^{2}}$$

where $\varepsilon_{n}$ is the energy of the n-th resonance level, $\Gamma_{n}$ is the half-width of the n-th resonance level, and N is the number of resonance levels. Moreover, if $E\notin \varepsilon_{n}\pm \Gamma_{n}$, then $T\left(E\right)=0$. This assumption is accepted, because outside the resonance level, $T\left(E\right)$ is negligibly small, while the Lorentz approximation overestimates $T\left(E\right)$.

The approximation (5) allows us to consider transport through a quantum well only for electrons with energies near the resonant level. Usually, this approximation is used to obtain a formula for the current. In the presented model, this approximation will also be used to estimate the concentration of electrons to consider inter-electron interaction. Let us express the LDOS at the “reservoir-channel” boundaries (2) using approximation (5):

(6) $$\rho_{s\left(d\right)}\left(z_{d\left(s\right)}\right)=\frac{m}{\pi \hbar^{2}}\frac{1}{\left|k_{d\left(s\right)}\right|}T\left(E\right)\approx \frac{1}{\pi \hbar }\frac{1}{v_{d\left(s\right)}}\overset{N}{\underset{n=1}{\sum }}\frac{\Gamma_{n}^{2}}{{\left(E-\varepsilon_{n}\right)}^{2}+\Gamma_{n}^{2}}$$

It follows from Formula (4) that the local electron density in the center of the quantum well can be written as

(7) $$\rho_{s\left(d\right)}\left(z_{w}\right)=\rho_{s\left(d\right)}\left(z_{d\left(s\right)}\right)\frac{v_{d\left(s\right)}}{v_{w}}\approx \frac{1}{\pi \hbar }\frac{1}{v_{w}}\overset{N}{\underset{n=1}{\sum }}\frac{\Gamma_{n}^{2}}{{\left(E-\varepsilon_{n}\right)}^{2}+\Gamma_{n}^{2}}$$

In addition to approximation (5), we will make one more assumption, that the width of the resonance levels is negligibly small compared to the thermal energy (at considered temperature T=300K). And since the order of accuracy of the linear approximation $f_{2D}$ (3) is determined by the magnitude kT, it is fair to consider $f_{2D}$ as a constant in the vicinity of the resonant level $\varepsilon_{n}\pm 2\Gamma_{n}$ (Figure 1, orange arrows).

Within the adopted assumptions of the model, we rewrite the equations for the current and electron density (1) as

(8) $$J\left(V\right)=\frac{qS_{m}}{\pi \hbar }\overset{N}{\underset{n=1}{\sum }}\left(f_{2D}\left(\varepsilon_{n}-U_{s}\right)\overset{\varepsilon_{n}+2\Gamma_{n}}{\underset{\varepsilon_{s}}{\int }}\frac{\Gamma_{n}^{2}}{{\left(E-\varepsilon_{n}\right)}^{2}+\Gamma_{n}^{2}}dE-f_{2D}\left(\varepsilon_{n}-U_{d}\right)\overset{\varepsilon_{n}+2\Gamma_{n}}{\underset{\varepsilon_{d}}{\int }}\frac{\Gamma_{n}^{2}}{{\left(E-\varepsilon_{n}\right)}^{2}+\Gamma_{n}^{2}}dE\right)\;n\left(z_{w}\right)=\frac{1}{\pi \hbar }\overset{N}{\underset{n=1}{\sum }}\frac{1}{v_{w}}\left(f_{2D}\left(\varepsilon_{n}-U_{s}\right)\overset{\varepsilon_{n}+2\Gamma_{n}}{\underset{\varepsilon_{s}}{\int }}\frac{\Gamma_{n}^{2}}{{\left(E-\varepsilon_{n}\right)}^{2}+\Gamma_{n}^{2}}dE+f_{2D}\left(\varepsilon_{n}-U_{d}\right)\overset{\varepsilon_{n}+2\Gamma_{n}}{\underset{\varepsilon_{d}}{\int }}\frac{\Gamma_{n}^{2}}{{\left(E-\varepsilon_{n}\right)}^{2}+\Gamma_{n}^{2}}dE\right)$$

where the values of $\varepsilon_{s}$ and $\varepsilon_{d}$ are determined by the following equations:

(9) $$\varepsilon_{s}=\left\{\begin{array}{ccc} \varepsilon_{n}-2\Gamma_{n} & \mathrm{for} & \varepsilon_{n}>2\Gamma_{n}\\ 0 & \mathrm{for} & 2\Gamma_{n}\leq \varepsilon_{n}\leq -2\Gamma_{n}\\ \varepsilon_{n}+2\Gamma_{n} & \mathrm{for} & \varepsilon_{n}<-2\Gamma_{n} \end{array}\right.\;\varepsilon_{d}=\left\{\begin{array}{ccc} \varepsilon_{n}-2\Gamma_{n} & \mathrm{for} & \varepsilon_{n}>-qV+2\Gamma_{n}\\ -qV & \mathrm{for} & -qV+2\Gamma_{n}\leq \varepsilon_{n}\leq -qV-2\Gamma_{n}\\ \varepsilon_{n}+2\Gamma_{n} & \mathrm{for} & \varepsilon_{n}<-qV-2\Gamma_{n} \end{array}\right.$$

In Equation (9), $U_{s}$ is taken as the zero level of potential energy. We will also assume that $U_{d}=-qV$.

Expanding the integrals in Equation (8) and using the relation for half-width $\Gamma_{n}=\hbar /\tau_{n}$, where $\tau_{n}$ is the average lifetime of an electron on the n-th resonance level, we obtain equations for the current and electron concentration of the compact model:

(10) $$\begin{array}{c} J\left(V\right)=qS_{m}\overset{N}{\underset{n=1}{\sum }}\frac{1}{\tau_{n}}\left(f_{2D}\left(\varepsilon_{n}-U_{s}\right)F\left(\varepsilon_{n}\right)-f_{2D}\left(\varepsilon_{n}-U_{d}\right)F\left(\varepsilon_{n}+qV\right)\right)\\ n\left(z_{w}\right)=\overset{N}{\underset{n=1}{\sum }}\frac{1}{v_{w}\tau_{n}}\left(f_{2D}\left(\varepsilon_{n}-U_{s}\right)F\left(\varepsilon_{n}\right)+f_{2D}\left(\varepsilon_{n}-U_{d}\right)F\left(\varepsilon_{n}+qV\right)\right) \end{array},$$

where $F\left(\varepsilon_{n}\right)$ is a function that describes the reduction in width of the resonance level at the bottom of the conduction band and is determined as

(11) $$F\left(\varepsilon_{n}\right)=\frac{1}{\pi }\left(arctg\left(2\right)-arctg\left(\frac{\varepsilon_{s}-\varepsilon_{n}}{\Gamma_{n}}\right)\right)$$

Thus, in the compact model, formulas for the current and electron concentration are functions of the energy characteristics of resonant levels. Therefore, to complete the construction of a compact model, equations for the energy and width of resonant levels are required. The half-width of the resonance level $\Gamma_{n}$ is determined by the structure of the RTD channel, while the energy of the resonance level is composed of the energy of the electrons in the absence of an external field $\varepsilon_{0}{}_{n}$, the energy of electron–field interaction, and the energy of the electron–electron interaction:

(12) $$\varepsilon_{n}=\varepsilon_{0}{}_{n}-kqV+q\phi ,$$

where $k=\left(z_{w}-z_{s}\right)/\left(z_{d}-z_{s}\right)$ is the antisymmetric coefficient of the RTD channel (equal to 1/2 for symmetric structures), and ϕ is the potential of the electron–electron interaction.

In the cogeneration model, the spatial distribution of the charge in heterostructures is considered using the self-consistent field method by introducing the average electron interaction potential, the self-consistent potential [20,21], into the Hamiltonian. In the developed efficient compact model, the interaction between electrons is considered using the potential ϕ. If ϕ is assumed to be equal to some voltage-independent constant and considered as a model parameter to be selected when validating the calculation results, then this approach will make it impossible to solve the inverse design problem. Therefore, in order to achieve the goals set by the authors, an estimate of ϕ is introduced into the model based on the fact [22] that the electron density in the RTS quantum well $n\left(z_{w}\right)$ is proportional to ϕ, which makes it possible to rewrite the resonance level energy as

(13) $$\varepsilon_{n}=\varepsilon_{0}{}_{n}-kqV+\frac{q^{2}}{C}n\left(z_{w}\right),$$

where C is the quantum capacitance of the channel.

Equations (10), (11) and (13) form a transcendental system of equations from which the current can be calculated. Thus, a compact model of current transport in an RTD channel is formulated, with the internal parameters being $\varepsilon_{0}{}_{n}$, $\Gamma_{n}$, C, $S_{m}$, k.

To better match the calculation results with experimental data, we supplement the compact model by considering the thermal current [23]:

(14) $$J_{tot}\left(V\right)=J\left(V\right)+e^{-\frac{a_{1}}{a_{0}}}\left(e^{\frac{V}{a_{0}}}-1\right)-e^{-\frac{a_{2}}{a_{0}}}\left(e^{-\frac{V}{a_{0}}}-1\right),$$

where $a_{0}$ is the thermal potential, and $a_{1}$, $a_{2}$ are the displacement potentials.

Also, the model considers parasitic resistance R as a sequentially connected load to an “ideal” RTD. Therefore, the final current through the RTD at the applied voltage $V_{tot}$ is determined by the following transcendental equation:

(15) $$J_{tot}\left(V_{tot}\right)=J_{tot}\left(V+J_{tot}\left(V\right)R\right)$$

### 2.2. Hysteresis and Negative Differential Conductivity Region

**Figure 2:**
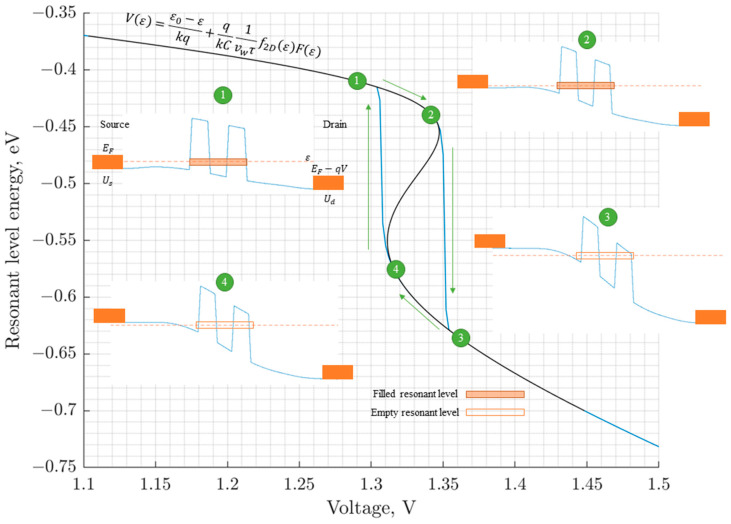
The mechanism of hysteresis formation in the current–voltage characteristics of a resonant-tunneling diode, black line for Equation (18), blue line for solution of (16).

The negative differential conductivity (NDC) region is of interest, among other things, because it is associated with the instability of the current flow through the structure channel [24], as well as the hysteresis phenomenon of the I-V curve, which, as shown below, is related to the instability of charge transport. Due to the rejection of the traditional cogeneration model in the approach proposed by the authors, the compact iteration of self-consistency provides an efficient calculation of interelectronic dissipative processes not only from a physical point of view, but from an algorithmic one; it establishes a “transparent” relationship between the electron concentration, current, and potential of interelectronic interaction. Figure 2 shows the developed compact model (in contrast to the widely used cogeneration models) that makes it possible to qualitatively and quantitatively describe the mechanism of hysteresis formation in the NDC region (Figure 2). 

The behavior of the RTS I-V curve in the NDC region can be explained as a manifestation of RTS bistability at near-peak voltages [25]. The essence of bistability is that the position of the peak point of the I-V curve at varying voltages depends on the sign of the voltage change, which leads to hysteresis [26]. The influence of inter-electron interaction on electron tunneling processes in the RTS quantum well is cited as a reason for such bistability [27]. The developed compact model can be applied to demonstrate how electron interaction specifically leads to the formation of a hysteresis loop in the NDC region.

For a qualitative explanation of the hysteresis phenomenon, it is sufficient to consider the case when there is only one resonant level in the system. Then, from Equations (10) and (13), we obtain the following transcendental equation:

(16) $$\varepsilon =\varepsilon_{0}-kqV+\frac{q^{2}}{C}\frac{1}{v_{w}\tau }\left(f_{2D}\left(\varepsilon \right)F\left(\varepsilon \right)+f_{2D}\left(\varepsilon +qV\right)F\left(\varepsilon +qV\right)\right)$$

At V near peak voltage, it can be assumed that $f_{2D}\left(\varepsilon \right)\gg f_{2D}\left(\varepsilon +qV\right)$, which gives an equation relating the voltage and the energy of the resonance level:

(17) $$\varepsilon =\varepsilon_{0}-kqV+\frac{q^{2}}{C}\frac{1}{v_{w}\tau }f_{2D}\left(\varepsilon \right)F\left(\varepsilon \right)$$

Equation (17) for ε is transcendental, but *V* can be expressed in the explicit form:

(18) $$V\left(\varepsilon \right)=\frac{\varepsilon_{0}-\varepsilon }{kq}+\frac{q}{kC}\frac{1}{v_{w}\tau }f_{2D}\left(\varepsilon \right)F\left(\varepsilon \right)$$

The numerical solution of Equation (16), compared with function (18), allows for a qualitative description of the mechanism of hysteresis formation (Figure 2).

Due to the non-monotonicity of the second term in Equation (18), the $V\left(\varepsilon \right)$ curve has a section with a negative slope, where dV/dε<0, corresponding to the NDC region on the I-V curve. This region is an area of instability, as Equation (17) has two solutions for *V* belonging to this region. Indeed, suppose the system is on a stable section with resonant level energy *ε* at voltage *V*. Then, when the voltage is increased by δV→0, the energy of the resonant level is equal:

(19) $$\varepsilon +\delta \varepsilon =\varepsilon +\delta V\frac{1}{dV/d\varepsilon }$$

The external voltage, which is the argument of the energy function of the resonant level (17), changes monotonically, which is determined by the test conditions. However, the calculation of the energy of the resonant level in accordance with Formula (19) contradicts this, since it leads to the curve 1-2-4-3 (Figure 2). Therefore, in this section, a hysteresis description of the dependence of the energy of the resonant level on the external voltage is required (cycle 1-2-3-4-1).

## 3. Materials and Methods

**Figure 3:**
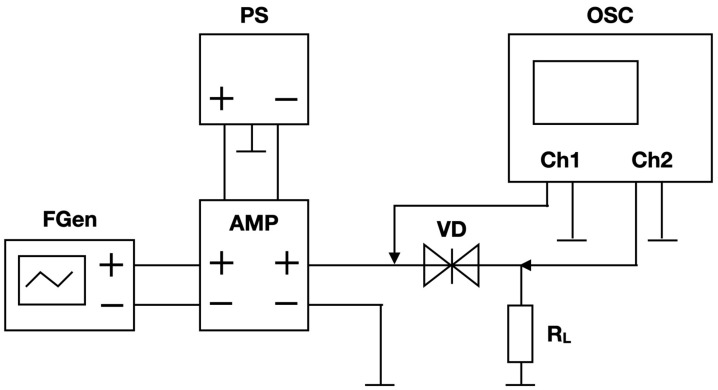
Measurement scheme. FGen—RIGOL DG4102 function generator, AMP—sound frequency amplifier based on TDA7293 microchip, OSC—Keysight MSOS804A oscilloscope, PS—RIGOL DP832 power supply, R_L_—10 Ohm load resistor, VD—tested RTD (DUT).

Figure 3 shows that the experimental investigation of RTD I-V curve hysteresis is based on the method of measuring the I-V curve of nonlinear elements, which involves simultaneous measurement of the instantaneous voltage value on the tested element (VD) and on the series load resistance (RL) under the action of alternating voltage at the input of the VD-RL circuit.

The investigated RTD samples were soldered onto prototype boards and installed in a fixture containing a load resistor *R*_L_, as well as connectors and contacts for connecting an AC voltage source and an oscilloscope. 

A function generator FGen was used as an AC voltage source, which generated a probing sawtooth waveform with a frequency $F_{G}=10\;\mathrm{kHz}$. To reduce the output resistance of the AC voltage source at the input of the VD-RL circuit, an audio amplifier AMP with a power of 140 W was used, powered by a PS power supply with an output voltage of ±30 V. The output resistance of the amplifier was 4 Ohm. Thus, the FGen and AMP circuit can be considered an ideal AC voltage source, the output resistance of which can be neglected when calculating the parameters of the RTD I-V curves.

**Figure 4:**
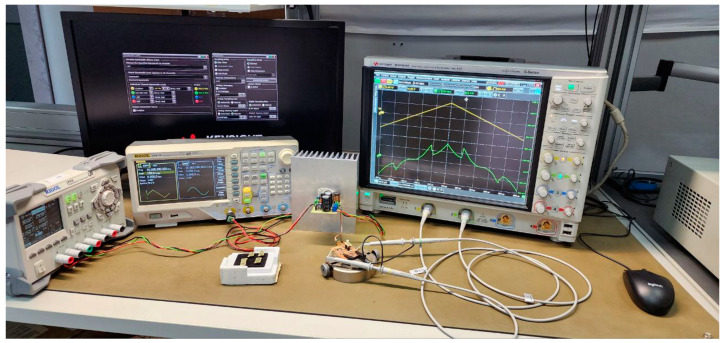
Stand for the analysis of the hysteresis of the RTD I-V curve.

Using the OSC oscilloscope, the value of the voltage *U*_Ch1_ (yellow oscillogram, Figure 4) at the input of the VD-RL circuit was recorded on channel Ch1, and the value of the voltage *U*_Ch2_ (green oscillogram, Figure 4) on the load *R*_L_ = 10 Ohm was recorded on channel Ch2.

Thus, the instantaneous value of the current through the diode VD can be defined as follows:

(20) $$i_{VD}\left(t\right)=u_{Ch2}\left(t\right)/R_{L}$$

The voltage across the diode at any given time can be determined as follows:

(21) $$u_{VD}\left(t\right)=u_{Ch1}\left(t\right)-u_{Ch2}\left(t\right)$$

As a result, the I-V curve of the RTD with increasing voltage will correspond to the dependence:

(22) $$i_{VD}\left(t\right)\;\mathrm{from}\;u_{VD}\left(t\right)\;\mathrm{at}\;t\in \left(0,\frac{T}{2}\right),$$

and with decreasing voltage:

(23) $$i_{VD}\left(t\right)\;oт\;u_{VD}\left(t\right)\;\mathrm{at}\;t\in \left(\frac{T}{2},T\right),$$

where $T=1/F_{G}$ is sawtooth voltage period, s.

The error estimation of hysteresis measurement consists, firstly, of the accuracy of the time scale of measuring $u_{Ch1}\left(t\right)$ and $u_{Ch2}\left(t\right)$, which is determined by the number of sampling points per period of the probing sawtooth voltage. In the conducted experiments, it was 2048 points per period. Due to the relatively large period of the probing sawtooth voltage T, the jitter of the FGen generator and the OSC oscilloscope, which are on the order of 10^−9^ s and 10^−13^ s, respectively, can be neglected. 

Secondly, the error is determined by the noise of the FGen generator and the AMP amplifier, as well as the inherent noise of the oscilloscope. The deviation of the effective voltage value of the generator caused by harmonic distortions at an output voltage of 1 Vpp, according to the technical description (standard deviation), does not exceed $\Delta U_{G}=2$ mV.

The magnitude of the amplifier AMP’s intrinsic noise, neglecting the flicker noise and fractional noise, can be estimated as the product of the effective value of thermal noise at the input of the amplifier with a resistance of $R_{in}=10\;\mathrm{kOhm}$ in the band B= 1 MHz (limited by the settings of the oscilloscope) at a temperature of *T* = 300 K and an amplification coefficient of AMP $G_{AMP}=27.6$. That is, $\Delta U_{AMP}=\sqrt{4kTR_{in}B}\times G_{AMP}\approx 0.5$ mV. 

The intrinsic noise of the oscilloscope when measuring in channel Ch1 with a vertical division price of 2 V/div is $\Delta U_{Ch1}=$ 50 mV (standard deviation).

The intrinsic noise of the oscilloscope when measuring in channel Ch2 with a vertical division price of 200 mV/div is $\Delta U_{Ch2}=$ 6.4 mV (standard deviation).

To reduce the influence of these errors on the results, averaging of measurements was introduced at each point $u_{Ch1}\left(t\right)$ and $u_{Ch2}\left(t\right)$, with *N* = 65,534 measurements.

Thus, according to the theory of processing indirect measurements, as well as the theory of statistical analysis of measurement results [28], the effective value of the total noise voltage when measuring the I-V curve of RTD can be estimated as follows:

(24) $$\Delta U=\sqrt{2{\left(\Delta U_{G}G_{AMP}\right)}^{2}+2\Delta U_{AMP}{}^{2}+\Delta U_{Ch1}{}^{2}+\Delta U_{Ch2}{}^{2}}/\sqrt{N}=0.36\;мB.$$

The parameters of the RTD experimental sample layers are presented in Table 1. Layers 6 to 10 represent a quantum region of RTS. Spacers serve to prevent the ingression of doping into the region from the transitional layers. Contact layers, buffer layers, and transitional layers have Si doping, with the concentration decreasing when approaching the undoped quantum region to minimize the number of dislocations in the quantum region.

The structure was grown using the method of molecular beam epitaxy on a semi-insulating GaAs substrate (layer 1) with 450 μm thickness. RTS had a mesa diameter of 20 μm after etching.

## 4. Results and Discussion

**Figure 5:**
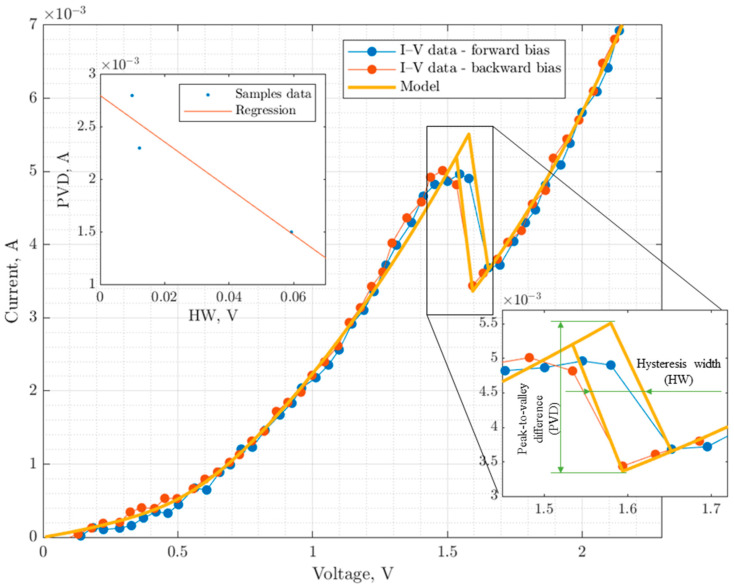
Comparison between simulated and measured RTD I-V curves.

To validate the developed model, a simulation of the mentioned RTD was performed, and a comparison was made between the calculation results and the measurements of the I-V curves (current–voltage characteristics). The analysis of the calculated and experimental I-V curves was conducted based on the following parameters: average error at different regions, peak and valley current errors, peak voltage error, PVD (Peak-to-Valley ratio), and hysteresis width. Figure 5 shows the validation results of the model.

The developed model allows for good agreement with the experiment both at the initial stage (with a relative error ∆ < 1.6%) and in the region of the Peak-to-Valley difference (PVD), while successfully validating the hysteresis width (HW). The deviation of the estimated HW from the measured value was 0.4%, and the deviation of the estimated PVD was 5% (see the caption in the bottom corner of Figure 3), with a relative error of measuring HW and PVD being 3.6%.

The high validation results lead to the conclusion that, for the first time, an effective and compact model has been constructed, capable of qualitatively and quantitatively describing the PVD of the RTD and the hysteresis phenomenon in the RTD I-V curves within the framework of stationary concepts of quantum-mechanical representations of processes in the channel, including dissipative processes of various natures in the RTD channel. The dominant influence on the bistable nature of the observed phenomena is the interelectron scattering in the device channel.

## 5. Conclusions

An effective current transport model has been developed for a nonlinear element based on a double-barrier heterostructure AlGaAs/GaAs for microwave signal converters, considering dissipative processes (including quantum-mechanical self-consistent calculation for interelectron interaction in the low-dimensional diode channel). The model is built on the basic ideas of so-called “compact” modeling, implementing decomposition principles that significantly simplify the qualitative and quantitative investigation and interpretation of the wave and dissipative properties of current transport in the channel, described by coupled quantum-mechanical equations. Such research and physical–mathematical analysis have, for the first time in world practice, allowed for the adequate prediction of the PVD region and hysteresis phenomena within a stationary model.

The algorithm’s time complexity has been reduced to a linear asymptotic estimate O(n) with a limited memory footprint, thanks to a typed vectorized approach to the software development process.

The validation of the model has confirmed its high accuracy not only in the initial region of the I-V curves (with a relative error ∆ < 1.6%) but also for characteristics that are traditionally predicted with low accuracy or only on a “qualitative” level without the possibility of quantitative evaluation. These characteristics include the following:

- Peak current (∆ < 1.7%);
- Peak voltage (∆ < 2%);
- Valley current (∆ < 0.01%);
- Hysteresis loop parameters (the error in loop width was ∆ < 0.4%, and in PVD—∆ < 5%).

This makes the developed compact model effective for modeling the operation of a wide range of devices with RTDs as nonlinear elements with operating points at different locations. From an engineering point of view, the developed model will enable the implementation of the target design principle, i.e., an approach that involves the individualization of the RTD’s construction for a specific device. This approach will provide the possibility of maximizing one or more performance indicators of the device based on the RTD, thanks to the possibility of simultaneous (or parallel) design of the nonlinear element and the final device. Due to its extremely low temporal and spatial complexity at high accuracy values, the potential for integrating the developed model into commercial professional systems for the automatic design of radio-electronic and optoelectronic devices based on RTDs is obvious.

## Figures and Tables

**Table 1:** RTD layer parameters.

Layer No.	Description	Chemical Composition	Conduction Type	Doping	Concentration, cm^−3^	Thickness, nm
1	Substrate	GaAs	SI	-		450 μm
2	Buffer layer	GaAs	i	-		0.2 μm
3	Buffer layer	GaAs	n	Si	4 × 10^16^	1.5 μm
4	Transitional layer	GaAs	n	Si	2 × 10^15^	30
5	Transitional layer	GaAs	n	Si	7 × 10^14^	50
6	Spacer	GaAs	i	-		2.26
7	Barrier		i	-		2.26
8	Well	GaAs	i	-		10.17
9	Barrier		i	-		2.26
10	Spacer	GaAs	i	-		2.26
11	Transitional layer	GaAs	n	Si	7 × 10^16^	50
12	Transitional layer	GaAs	n	Si	2 × 10^17^	30
13	Contact layer	GaAs	n^+^	Si	4 × 10^18^	50
14	Contact layer	Gradient InGaAs 0.05 to 0.5	n^+^	Si	4–5 × 10^18^	50
15	Contact layer	In_0.5_Ga_0.5_As	n^+^	Si	5 × 10^18^	20

## Data Availability

Not applicable.
